# A qualitative study on the views of experts regarding the incorporation of non-health outcomes into the economic evaluations of public health interventions

**DOI:** 10.1186/s12889-015-2247-7

**Published:** 2015-09-24

**Authors:** Ghislaine APG van Mastrigt, Aggie TG Paulus, Marie-Jeanne Aarts, Silvia MAA Evers, Adrienne FG Alayli-Goebbels

**Affiliations:** Department of Health Services Research, CAPHRI School of Public Health and Primary Care, Maastricht University, P.O. Box 616, 6200 MD Maastricht, The Netherlands; Trimbos Institute, Netherlands Institute of Mental Health and Addiction, Utrecht, The Netherlands; Institute for Health Economics and Clinical Epidemiology, University Hospital of Cologne, Gleueler Strasse 176-178, 50935 Cologne, Germany

**Keywords:** Non-health outcomes, Economic evaluation, Expert views, Public health interventions, Patient-reported outcomes measurements, Qualitative research

## Abstract

**Background:**

Public health interventions can impact a broad number of outcomes, including both health and non-health outcomes (NHOs). However, although it is often acknowledged that it’s important to take NHOs into account in economic evaluation studies, in practice these are often neglected. To address this issue, our study investigated expert views regarding the incorporation of NHOs into the economic evaluations of public health interventions, by means of a qualitative study.

**Methods:**

A purposive sampling method was used to recruit the experts in the field of health economics and/or public health for this study. Twenty-two semi-structured interviews were held. After recording, the interviews were transcribed verbatim and entered in Nvivo. The data was analyzed using a thematic analysis to identify all important themes mentioned by the experts. Data collection and analysis was continued until saturation was reached. Multiple coding and validity checks were performed to further strengthen the rigour of our methodology.

**Results:**

Based on the expert interviews, the following overarching themes were identified; Theme 1: NHOs on the individual level, direct social level and societal level. Theme 2: Pros and Cons regarding the development of a new instrument to measure NHOs. Theme 3: The most important requirements for a new questionnaire to be developed for measuring broader outcomes. Theme 4: Alternative methods which could be used for measuring and valuating NHOs in economic evaluations for public health.

**Discussion:**

Our research findings indicate that the importance of NHOs and the need to measure them are universally accepted. Most of the experts acknowledge the importance of measuring broader outcomes and support the development of a new instrument to measure these. The experts, who do not support the development of a new instrument, question its usefulness and feasibility; i.e., they are not sure whether it is possible to valuate NHOs. Furthermore, experts have strong and sometimes conflicting views on the specific requirements of a new instrument to be developed for measuring NHOs. They did not identify a single preferred alternative method for measurement and valuation.

**Conclusions:**

Most experts find a wide range of NHOs important and are in favor of developing a new instrument for identifying and measuring NHOs. Hence, an open discussion needs to be initiated with experts and other stakeholders about which steps need to be taken to move forward.

## Background

Although attention is increasing in the UK and elsewhere on incorporating non-health outcomes (NHOs) as well as health-related outcomes in the economic evaluations (EE) of Public Health (PH) interventions [1], incorporating NHOs is not yet common practice [2–4]. The World Health Organization defines PH as all organized measures (whether public or private) to prevent disease, promote health, and prolong life among populations (http://www.who.int/trade/glossary/story076/en/). PH interventions include both simple interventions, which target only individuals, and more complex multimodal or multilevel programmes, which address policies or characteristics of the physical environment. Examples are, for instance, interventions that seek to control infectious diseases, regulations that ensure the production and sale of safer and healthier foods, the promotion of breast-feeding, the introduction of bicycle lanes and crime prevention measures. NHOs in this study were defined as outcomes that are not captured by the five dimensions included in the EQ-5D (mobility, self-care, usual activities, pain/discomfort and anxiety/depression). NHOs that may result from PH interventions are often described as broader outcomes or outcomes which are not captured by standard measures of health outcomes. Examples of NHOs include, for instance; ‘’increased health knowledge”, ‘’increased participation”, ‘’increased safety in the neighborhood” and ‘’educational success”.

There are four important reasons for not taking NHOs into account in the EEs of PH interventions:

First, it is still unclear which NHOs are relevant for measurement and inclusion in an EE of a specific PH intervention [5, 6].

Second, it can be difficult to identify and measure all relevant NHOs in EEs of specific PH interventions, as the outcomes of PH interventions do not always occur at the same operating level as the intervention. For instance, these interventions can influence not only targeted individuals (the first level) but can also have an impact on other people in the community, including some who are not directly targeted by the study (the second level), or even on society at large (the third level) [7]. Although the National Institute for Health and Care Excellence (NICE) [8] recommends incorporating NHOs in EEs of interventions aimed at improving both health and NHOs in the public sector and in other sectors in its reference case, recent NICE guidelines of PH interventions reveal that this is still not always common practice (PH50 and PH53) [9, 10].

Third, existing questionnaires for measuring NHOs do not meet all needs. For instance, they include only NHOs within a few domains and on a single level: like Ascot [11], which is designed to measure the individual social care-related quality of life, or questionnaires that have been designed for specific populations, e.g. the ICEpop CAPability measures, ICECAP-A for Adults [12], ICECAP-O for older people [13] and ICECAP-SCM for use in end of life settings [14].

Fourth, traditional EE methods have limitations regarding whether and how NHOs can be measured and valuated as an outcome [4, 15]. The most commonly used EE methods of PH interventions are cost-effectiveness analyses (CEA), cost-utility analyses (CUA) and cost-consequence analyses (CCA) [3, 4, 16]. In a CEA, outcomes are measured in natural units - for example, life years saved or infections averted. In a CUA, outcomes are expressed as quality-adjusted life years (QALYs) and utilities are mostly determined by means of off-the-shelf instruments that measure health-related quality of life, such as EuroQoL (EQ-5D) [17]. In a CCA all relevant outcomes can be taken into account, although they are presented in a non-aggregated way. Several alternatives have been proposed, which could address the limitations of traditional EE methods: (1) CUA with an ‘’expanded QALY”, which incorporates dimensions other than health, (2) a CUA or CEA using a ‘’multi-sectoral approach”. In the latter approach, the inter-sectional costs and effects are simultaneously captured and adjusted for budgets and resources that are allocated by various ministries [18]. (3) Using cost benefit analysis (CBA), in which both cost and consequences are expressed in monetary units, and (4) multi-criteria decision analyses (MCDA) using various criteria (e.g. incremental cost-effectiveness ratio, budget impact or severity of disease), which are relevant for setting priorities. However, currently these alternative EE methods are seldom applied in these settings [3, 4].

The above-mentioned challenges regarding the identification, measurement and valuation of NHOs in the EE of PH interventions are increasingly acknowledged [3, 19, 20], and the importance of incorporating the benefits of NHOs in the EEs of PH interventions is highlighted [3, 21, 22]. However, it is still unknown what health economics and public health experts think is the best way to move forward: either developing a new broader questionnaire, or using existing methods to identify, measure or -valuate NHOs. Furthermore, it is unknown which outcomes NHOs experts find relevant, i.e. which need to be taken into account in EEs of PH interventions. The aim of the current study was to investigate the views of leading experts on these topics.

## Methods

### Recruitment of participants

A purposive sampling method was used to recruit the experts [23]. They were identified based on diversity of expertise and affiliations, years of work experience and recent publications in the fields of public health and health economics. All experts were contacted by email by one of the researchers (MJA). The invitation to participate in the study contained the background and the purpose of the study, together with a proposed time and date for an appointment. If there was no reaction to this initial e-mail, experts were contacted by telephone. In a second wave, an additional group of experts was contacted; these were identified by means of snowball sampling (by identifying respondents who are then used to refer researchers to other respondents) [24]. Data collection continued until saturation was reached (i.e. until no new themes emerged from the data) [25]. In order to obtain insight as to whether new (sub)-themes appeared after the interview, the interviewer (MJA) compared field-notes made during each interview with previous interviews. After 21 interviews, no new (sub)-themes were identified, and data collection/recruitment was stopped. The response rate was calculated by dividing the number of experts who refused to participate by those who were willing take part in the study.

### Interviews

Interviews were considered the most appropriate method for collecting data, to gain insights into the views and experiences of individual experts. The semi-open structure of the interviews allowed interviewees to elaborate on particular areas which they regarded as important. A semi-structured interview protocol containing a set of open-ended questions for gathering expert views was used to discuss the identification, measurement and -valuation of NHOs in the EE of PH interventions (Appendix 1). We explained that we define NHOs as outcomes that are not captured by the five dimensions included in the EQ-5D (mobility, self-care, usual activities, pain/discomfort and anxiety/depression). The EQ-5D was used for this as it is one of most frequently used preference-based questionnaires for measuring health in economic evaluations. The interviews were audio-recorded, transcribed verbatim by two persons and were verified for validity against the recording (MJA and experts). Interviewees were asked by mail to verify the classification of background characteristics, their work experience in number of years and the content of the transcribed interview.

### Analysis

A thematic analysis was conducted using NVivo (version 9.0). The coding process involved five phases in creating established, meaningful (sub-)themes and headings. First, two researchers (GvM and JS) read through the transcript to become familiar with the data. Second, transcripts were read more thoroughly, and the initial codes of themes were applied to sections, sentences or words as they emerged in the data. This was done using the coding protocol based on the field notes made by the interviewer (MJA) (Appendix 2). Third, sub-themes and headings were identified, and the coding protocol was adapted if necessary (Appendix 2). Finally, phases two and three were repeated and if needed new sub-themes and headings were added [26].

Disagreements in coding between the two researchers were discussed between two authors (GvM with AAG, or AP, or SE) resulting in final coding of (sub) themes and heading(s).

Multiple coding was performed to investigate the inter-rater reliability among the researchers who performed the coding [27]. For this the percentage of disagreement was calculated for each of the interviews.

Furthermore, selective data use was also investigated in relation to the impact of specific interviews on the classification of the most discussed NHO topics in the first theme. The analyses were performed with and without the inclusion of two experts who reported many of the NHOs.

The results are presented for every theme, including a description of results and illustrative quotes. We choose to use a (semi-) quantified approach to present the results for each theme [28]. Presenting simple counts of themes can help readers gain a sense of how widespread a particular view is among the experts interviewed [29]. However, these counts do not say anything about the importance of each theme. For the clarity of the description results, all quotations are displayed in italics, with double commas.

### Ethical considerations

This research was conducted according to the principles of the Helsinki declaration. It was exempted from ethical review under the scope of the Medical Research Involving Human Subjects Act (WMO), which protects study participants in the Netherlands. Participation was voluntary and the interviewees were free to stop the interview at any time. Before the interviews began, the purpose of the study was explained again and informed verbal consent for participation in the study was obtained. All experts were asked for informed consent to use their names in the paper. As six of the experts preferred to stay anonymous, we did not refer to any expert by name when quoting, but named them in the acknowledgement section. The background information of the study population is provided at such a level of detail that which expert said what cannot be traced.

## Results

### Interviews

We selected a total of 23 experts; sixteen of these responded to our original invitation for an interview and the other six were identified by snowball sampling. Only one of the selected 23 experts refused to participate in the study for an unknown reason; he did not respond to any emails we sent to him and could not be reached by telephone. Nevertheless, the response rate for this study was very high (95.7 %). Twenty of the experts were interviewed in person, and the other two were interviewed by phone. The duration of both the telephone and face to face interviews was approximately one hour each.

All interviews were conducted in English by one researcher (MJA) between December 2011 and May 2012. There was an informal atmosphere during the interviews, with an occasional interruption as all experts were in their office at the time the interview took place. Two research assistants (IB, ES) transcribed the taped interviews. All transcripts were checked for validity by one researcher (MJA) and fourteen were also checked by the experts themselves; these checks did not result in any significant content changes. The inter-rater reliability was low; on average per interview 21 % of the coded items were comparable between the two researchers (GvM, JS). This was related to the fact that during coding by the second reviewer the coding protocol was adapted; items scored by JS in categories 2.1 and 4.0 were coded, respectively, in Section 2.2 and 2.1 by GvM. Furthermore, LS used two additional coding categories: ‘’evaluation of future challenge” and ‘’usefulness of the instrument”, which were recoded after consensus meetings of the authors into, respectively, categories 2.1 and 4.0 (see Appendix 2 for coding protocol and adaptations).

### Background sample

At the time the interviews took place, only two experts were employed by a private company. All 22 of the experts interviewed were experienced scientists; among them were fourteen professors, four senior researchers, three directors and one associate director, all affiliated with public organisations (universities *n* = 14, institutes *n* = 6, university hospitals *n* = 2). Ten of our experts were members of the advisory councils involved in PH decision-making. In addition, six of them worked in one of the following institutions: NICE, the Trimbos Institute (the Netherlands Institute of Mental Health and Addiction), the National Institute for Public Health and Environment (RVIM in the Netherlands) and the Centre of Excellence in Intervention and Prevention Science (CEIPS in Australia) on research directly supporting government policymaking. The 22 experts were working in the field of health economics (*n* = 9), public health (*n* = 5) or both (*n* = 8). However, According to the date of their first scientific publication in PubMed, these experts had on average 22 years of work experience. Eleven of them were English, seven Scottish, three Dutch, and one was Australian.

Four overarching themes represent the essence of the interviews;

1. Non-health outcomes (NHOs) mentioned by the experts (Theme 1), categorized in three subthemes: on an individual level, on a direct social level and on the societal level.
2. The view of the experts for or against the development of a new instrument to measure NHOs (Theme 2).
3. The view of the experts on the most important requirements for a new questionnaire to be developed for measuring broader outcomes (Theme 3).
4. The experts’ views on alternative methods which could be used for measuring and valuating NHOs in EEs for PH (Theme 4).

The following four sections expand on these four themes and the different subthemes that were identified.

- Theme 1: Non-health outcomes (NHOs) mentioned by the experts

**Graph 1:**
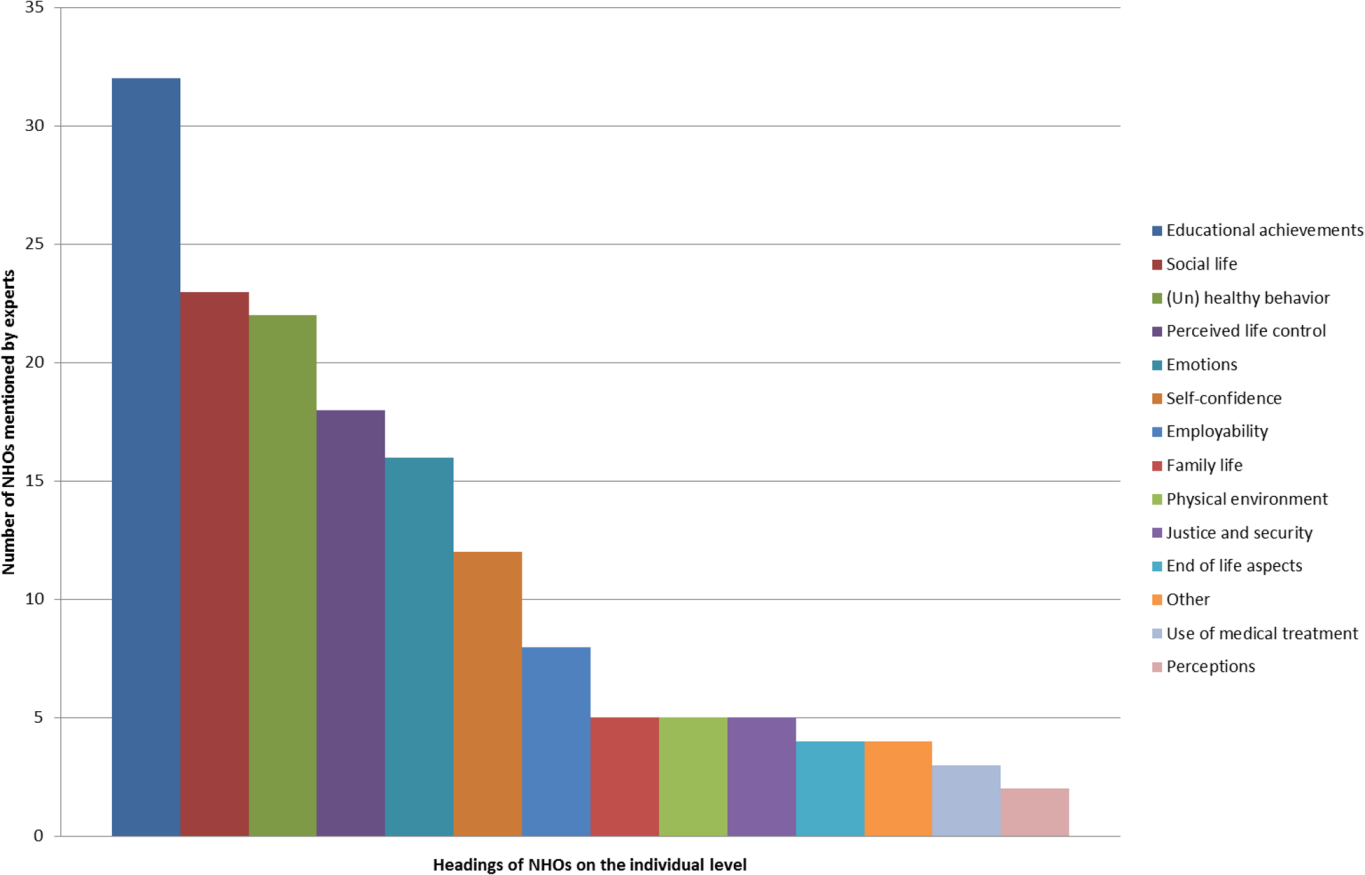
Displays the number of experts who talked about the different headings of the individual NHOs

More than 300 specific NHO quotes were identified in total. These were categorized on three different levels. First, quotes are described concerning NHOs which focus on the individuals targeted by the PH interventions. Expert quotes on this subtheme were identified under fourteen different headings (Graph 1). The most frequently mentioned NHO topic in the subtheme of individual NHOs was educational achievements, which included quotes on ‘’*educational output”* and ‘*’an increase in Intelligent Quotient (IQ)*”. The second heading in the subtheme is about behaviour and includes quotes on healthy behavior, such as ‘*’frequency of walking*”, but also quotes on unhealthy behavior, such as ‘*’alcohol and/or drug abuse*”. The third heading in this subtheme is related to aspects of social life, and includes quotes on ‘*’frequency of social contacts”, ‘’going out with friends”* etc. Perceived life control includes NHOs on ‘*’freedom’*’ and ‘*’autonomy*”. The other headings mentioned by experts and included in this subtheme were emotions (including quotes on, for instance, *‘’a sense of satisfaction”)*, self-confidence *(*quotes *on ‘’self-esteem, ‘’self-efficacy”)*, perceptions, family life *(*quotes *on “have a partner”)*, employability *(*quotes on *‘’ability to work”),* physical environment (quotes on ‘*’insulation of houses”*, *‘’asbestosis”*), use of medical treatment (quotes on ‘*’medicine use”*), end of life aspects *(*quotes *on ‘’being prepared for death”*) justice and security (quotes on ‘*’new security mechanisms for houses*”) and other (quotes on ‘’*sexual dysfunction”*). (For all sample quotes of NHOs in this subtheme see Appendix 3: Table 1).

**Graph 2:**
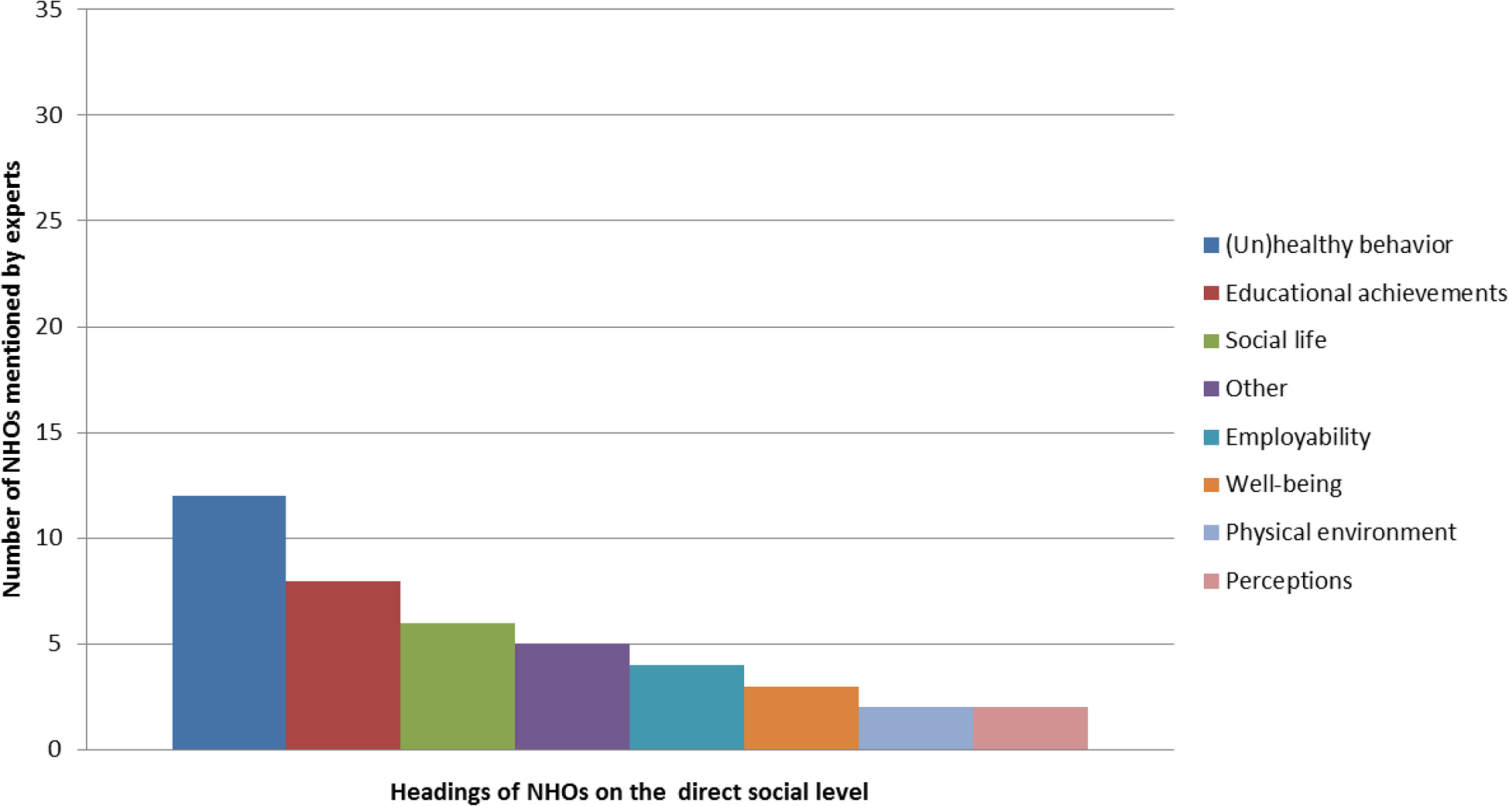
Displays the number of experts who talked about the different headings of the direct social NHOs

The second subtheme, ‘direct social level’, defined NHOs that have an impact on individuals who do not take part in the PH interventions themselves but are family members or classmates of those targeted individuals. These quotes were categorized in 8 headings (Graph 2). The three most frequently mentioned headings at the direct social level were educational achievements, (un) healthy behavior and social life aspects. The first heading contains quotes like ‘*’increase of concentration levels of the class” and ‘’impact on truancy rates in classes’*’, and the second heading, (un) healthy behavior, contains quotes like ‘*’çooking for the family and eating properly”.* Aspects of social life includes quotes on, for example, *‘’social exclusion”*. Other less frequently mentioned headings were well-being, employability, physical environment, perceptions (e.g. *‘’people’s perceptions of the neighborhoods”* and the remaining heading, other (for sample quotes of all NHOs in this subtheme see Appendix 3).

**Graph 3:**
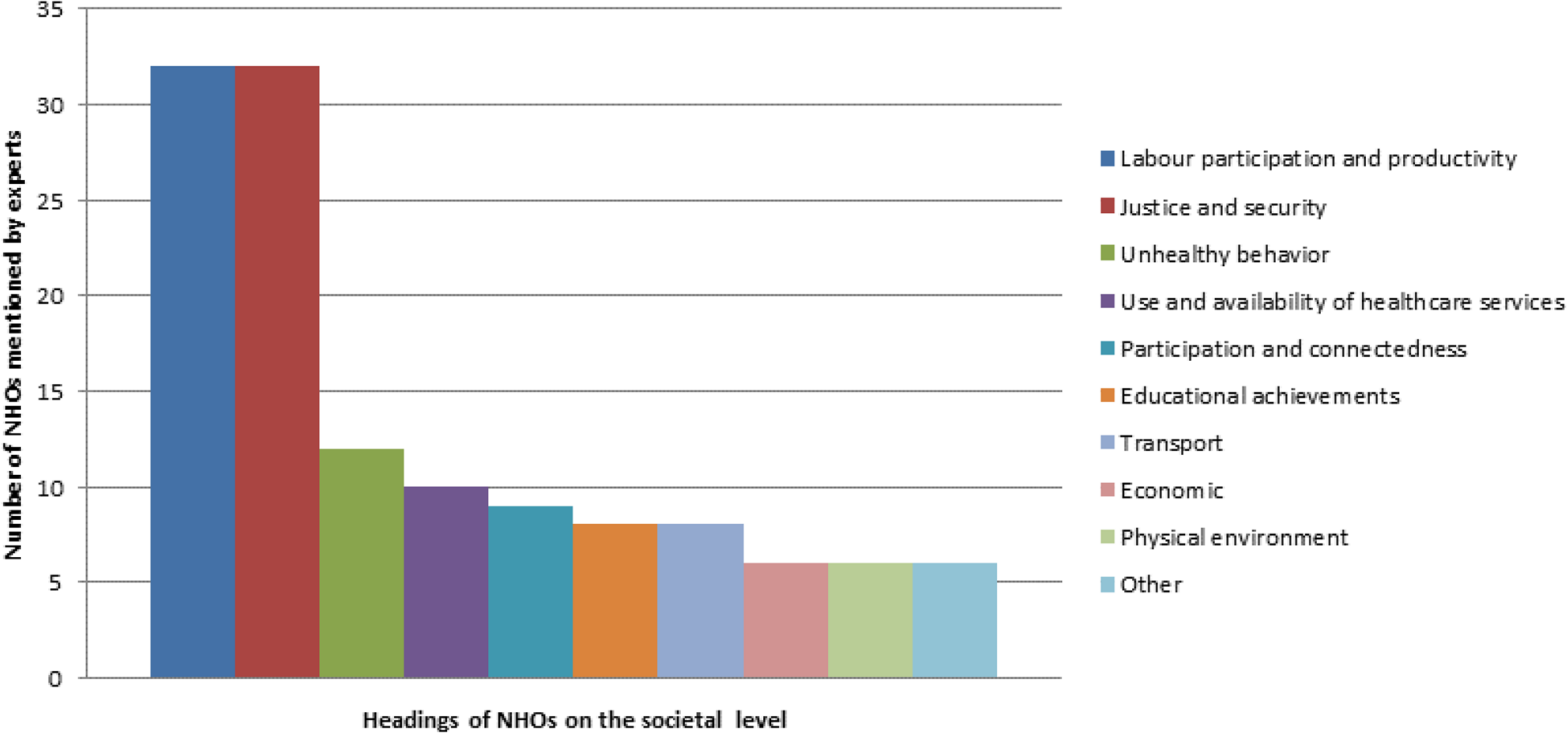
Displays the number of experts who talked about the different headings of the societal NHOs

The third subtheme (Graph 3), societal NHOs, which represents all NHOs which can impact society as a whole, was dominated by the two headings: Labor participation and productivity, and Justice and security. NHOs mentioned in this subtheme were, for instance, *‘’lack of employment”*, ‘*’reduction of number of teachers”* for the first heading, and ‘*’crime related to alcohol consumption*” for the second (For all headings and sample quotes of NHOs in this subtheme, including the eight not mentioned here, see Appendix 3, Table 1).

After the data were analysed it appeared that two of the experts reported 15 % (*n* = 50) of all quotes in theme 1. When the results of these two experts were excluded from the analysis, all of the ‘main’ NHO headings previously discussed were identical, except for one heading, ”availability and use of healthcare services at the societal level”. This was replaced by one of three other headings: educational achievements, Transport and Economic.

- Theme 2: Pros and cons regarding the development of a new questionnaire for measuring broader outcomes

Fourteen (64 %) of the 22 experts interviewed were in favor of developing a new instrument for measuring broader outcomes. Among these were all the experts classified in the public health group, five of the eight classified in the health economics group and three out of the six with backgrounds in both health economics and public health. The seven other experts (31 %) don’t think it is possible to develop such a questionnaire. Three of these were classified as health economists, and the four others were classified in the group ‘’both.” One expert did not talk about this topic at all.

Most of the pro arguments were related either to the importance of NHO measurement (*n* = 10) or to the need for having a new questionnaire (*n* = 4). A public health researcher highlights the importance of measuring NHOs for him/her as follows: ‘*’We are missing an awful lot, and that's not a criticism of our research field; it is criticism of the work that I do myself.*” Another expert expressed the need for developing a new instrument as follows “*I would say it is very important, so, a) it is feasible, b) it is necessary and c) it is desirable.*”

Four of the seven experts who are against developing such a questionnaire doubt the feasibility of measuring all possible NHOs with a single instrument. One health economist stated his/her doubt about integrating the measurement of a variety of NHOs into one instrument as follows ‘*’The sheer number of different things which one might need to measure is going to rule out a single instrument, I think. A single instrument would be too complicated.”* Other arguments against related to the -valuation of NHOs (*n* = 3). One expert noted that he/she thinks the main problem is the -valuation of NHOs and how these -valuations can be compared with health outcomes: *‘’Having the instrument, it doesn't solve the valuation problem, does it? It does not get you away from the fact that you need to be able to trade health and non-health.”* Three experts state that a questionnaire for broader outcomes would not be used to inform allocation decisions because policy decisions are based on CUA, which normally does not include these broader outcomes. One expert mentioned that British policymakers will not base their decisions on only monetary values, but on cost per QALY as a study outcome. *‘’We could value NHOs using cost benefit analysis or contingent –valuation, but then our decision-making body NICE, they don't value these outcomes, they want everything in QALYs.”*

- Theme 3: Views on the requirements for a broader outcome measure

Various aspects that need to be taken into account in the design phase of a new instrument for measuring NHOs are mentioned by the experts, including the choice of the method used for identifying domains/NHOs (Subtheme 1), the boundaries of the questionnaire (Subtheme 2), the level of NHO measurement (Subtheme 3), the length of the questionnaire (Subtheme 4), the preferred concepts (Subtheme 5), the -valuation of NHOs measured in the newly developed questionnaire (Subtheme 6) and how to incorporate the outcomes of such a new instrument into existing economic analysis methods (subtheme 7). These seven subthemes will be discussed in detail in the following sections:

- (Subtheme 1) Choice of method for identifying NHOs/domains

According to six experts the best ways to identify all relevant NHOs are to perform a literature review or choose a relevant theory or framework to start with. For instance, one public health expert suggested using Maslow's hierarchy of needs as starting point for identifying all relevant NHOs. ‘*’Use a framework, like health or Maslow’s theory as starting point, to identify a hierarchy of things that people want from life.”* Six other experts think it is better to ask the general public, experts or stakeholders to identify the important NHOs. A health economist thinks a stakeholder should be the person designated to identify the (dis) advantages of PH interventions. ‘*’Ask the stakeholder to identify the range of possible benefits and dis-advantages in different sectors.”*

- (Subtheme 2) Boundaries of the questionnaire

Eleven of the experts prefer broad boundaries for such an instrument. One of them stated, ‘*’Catch everything”,* which can be interpreted as being comprehensive. Another public health expert expressed his/her doubt regarding the possibility of setting the right limits. ‘*’Well, I think it should be as broad as possible but I don't think that means it could cover everything because that would not be possible.”* In contrast to the view that it needs to be as broad as possible, the majority of experts consider the measurement of NHOs to be context-specific (*n* = 12). More specifically, they state that the measurement of NHOs depends on the intervention being -valuated (*n* = 9), on the provider of the subsidy (*n* = 5) or on the setting (*n* = 3). One of the experts says that it is even ”*Impossible to make a generic instrument which can be used in any situation”* because the instrument is dependent on the provider of the subsidy and on the type of intervention being evaluated: *‘’So there wouldn’t be a specific set of things I’d always do, it would depend on the contact and it would depend on the intervention.”*

- (Subtheme 3) Level of NHO measurement

Almost fifty percent of the experts preferred NHO measurement on an individual (*n* = 11) and on a direct social level (*n* = 10). One-third (*n* = 7) think a future instrument should include items on a societal level as well. Of these experts, five are in favor of measuring on all three levels. The six others prefer measuring on two levels, either individual and direct social (*n* = 4) or individual and societal (*n* = 2), or prefer to focus on measurement at a single level (*n* = 1). One expert makes a very clear statement regarding his/her preference for not restricting the measurement of NHOs to one level, *“As soon as you focus on only one level I think you miss half of the outcomes.”* Another expert stated the need for measuring both individual and direct social NHOs as follows: “*It should capture both of those individual and relational dimensions, because they are both significant in understanding the nature of the problem.”*

- (Subtheme 4) Length

Another subtheme mentioned frequently is the desirable length of a future instrument. Six experts state that it needs to be either ‘’*practical*”, ‘’*manageable*” or ‘’*short.*” One of them is in favor of a short questionnaire which can be filled in on a smart-phone app, and says: ‘*’Ten questions … which you can do on an I-phone”*.

- (Subtheme 5) Preferred contents

In relation to the contents of a new questionnaire, experts talked about the concepts preferred for inclusion. These are happiness or well-being (*n* = 5), quality of life (*n* = 4), capability (*n* = 3), and function or utility (*n* = 1). A firm statement of a public health expert on his/her preference for measuring quality of life instead of health-related quality of life is, *‘It is just a general quality of life measure. That is all we need.”* One of the experts thinks it is possible to incorporate four concepts into one questionnaire, ‘*’I think it's definitely something around an instrument that captures quality of life and capability, utility and function, I suppose.”*

- (Subtheme 6) Evaluation of development

Nine experts made statements regarding how the design of a new questionnaire needs to be done. For instance, they find it relevant to check the psychometric properties (like validity, reliability) and responsiveness of a new instrument. One of them says, ‘*’It needs to be validated using focus groups, cognitive debriefing, test and retest, reliability and all of those sorts of things that go into good questionnaire design.”* Another expert mentioned that an instrument needs to be sensitive or otherwise it is useless, ‘*’But its sensitivity is crucial, isn't it; it has to be sensitive to change, as a result of some of these other activities, and if it isn't sensitive to change, then we can't use it to measure outcomes. Then it is just a wish list.”*

- (Subtheme 7) Incorporation of NHOs in economic analysis

Another topic which the experts talked about was how the outcomes of a newly to be developed NHO questionnaire need to be incorporated in the existing methods of performing economic analysis. Four experts mentioned that the threshold for a PH intervention being considered cost-effective needs to be adjusted. One of them noted, *‘’Just as we need a threshold for cost per QALY, you need another threshold that tells you something about how you move between health and non-health.”* Another expert mentioned the problem of having non-health outcomes besides health outcomes and the presentation of these data in a cost-effectiveness plane as follows: *‘’If you think of the cost effectiveness plane, you then would have another dimension, wouldn’t you? You would have the cost dimension, the health-related dimension and the non-health related dimension. Is that right?”*

- Theme 4: Views on the use of alternative methods for the measurement and valuation of NHOs.

**Fig. 1:**
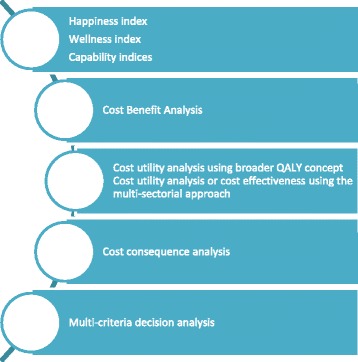
Alternative methods for measuring and evaluating NHOs

Most of the experts (16 out of 22, 73 %) expressed their view on at least one of the alternative methods for the measurement and -valuation of NHOs (Fig. 1). In the first subtheme, alternative methods for measuring NHOs using existing questionnaires, like the Happiness, Wellness and Capability Indices will be discussed. In the second subtheme the use of another type of economic evaluation, the cost benefit analysis, will be discussed. In the third subtheme the use of cost utility analysis using a ‘’broader QALY approach” as a measure of effects, and cost utility analysis or cost effectiveness analysis using an ‘’multi-sectorial approach” will be discussed. Finally, the cost consequence analysis and multi-criteria decision analyses (MCDA) were identified as alternative methods (Subthemes 4 and 5).

- (Subtheme 1) Happiness, Well-being and Capability Indices

Four of the experts talked about the Happiness Index or Wellbeing Index for measuring NHOs, and eight experts mentioned capability indices as an option, although only one of them is in favor of using this method. One of the experts notes that it is difficult to measure capabilities by saying; *‘You’re asking, are you capable of doing this, rather than do you do it or not, I think it is difficult to ensure that the person who is answering, is answering it right.”*

- (Subtheme 2) Cost benefit analysis (CBA)

The use of cost benefit analysis for incorporating non-health outcomes in economic evaluations was mentioned by six experts, who were all in favor of using this method. For example, if you are using CBA, you don’t need to worry about boundary issues - as one of the experts noted, *‘’You don’t have to worry about a complete list of NHOs.”* One other expert thinks that policymakers like to have broader outcomes expressed in monetary value*s. ‘’When you attach monetary values to NHOs you begin to communicate to an audience that might not otherwise listen.”* Opponents of this method argue that the individual preferences which are mostly used in CBA do not represent real world decisions. An expert stated that societal preferences are the basis of a public-funded health care system and not those individual preferences which are measured in CBA. *‘’The drawback of using individual preferences is that these individual preferences aren’t expressed through the market.”* Another drawback mentioned is that CBA requires considerable investment in labor as it needs to be tailored for every single intervention.

- (Subtheme 3) CUA using a broader QALY concept, or CEA or CUA using the ‘’multi-sectorial approach”

Most of the experts who mentioned the CUA using a broader QALY concept don’t think it is a solution for the measurement of NHOs. As one of them stated, ‘*’I mean ideally we need a measure that captures everything. Like a beyond the QALY, like a social, a societal QALY something, so the QALY we use just in health care but if there was a QALY equivalent which was across all the other budgets, that's the ideal, the theoretical ideal. That's the ideal, but I can't see how it will ever… I can't see how it will get there.”* An alternative method that can be used to measure and -valuate non-health outcomes can be the use of a CEA or CUA incorporating a multi-sectorial approach, instead of using a societal approach. One expert stated the potential advantage of using this method as follows; ‘*’What we need to know, is what are all the impacts on the different sectors, and then we need to add up, who's a gainer, who is a loser; do the gains compensate the losses?”*

- (Subthemes 4 and 5) Cost of consequence analysis (CCA) or multi-criteria decision analyses (MCDA)

CCA and MCDA were also mentioned by the interviewed experts as possible methods which could be used, as these methods can take into account broader outcomes of public health interventions.

## Discussion

### General findings

To our knowledge this is the first study, which has investigated the views of leading scientific experts in the field of health economics and public health on the measurement of NHOs for the economic evaluation of public health interventions. As the views of experts with different backgrounds were reported in this study, an objective overview of current thoughts on this topic is given from different perspectives. Most of the experts acknowledge the importance of measuring broader outcomes and support the development of a new instrument to measure these. The experts who are against developing a new instrument argue that they doubt the feasibility of developing a single instrument for measuring all NHOs at once; they state that -valuating NHOs is very difficult and that such an instrument would not be used to inform reimbursement decisions on public health interventions. Both proponents and opponents of the development of a new instrument have explicit views on the requirements a new questionnaire instrument should fulfill; on one hand it needs to have broad boundaries and therefore include different NHO levels, but on the other hand they also think it should be as short and manageable as possible. They discussed the usefulness of existing questionnaires for measuring NHOs, like the Happiness, Wellness, and Capability Indices. In addition, they talked about alternative EE methods that incorporate non-health outcomes, like CBA, and the use of CEA or CUA in combination with a ‘’broader QALY” or multi-sectorial approach, but also CCA and MCDA. As the uses of these existing questionnaires and alternative EE methods have certain advantages and disadvantages, none of these methods was preferred over the others by our experts.

### Individual, direct social and societal NHOs

PH interventions can generate very broad costs and benefits and are often directed at populations or communities rather than at specific individuals [3, 6]. Therefore, it is important to gain insight into which NHOs are relevant for measuring not only at the individual but also at the community or direct social and the societal or population level. The variety of NHOs mentioned at all three levels by the experts indicates the broad scope of NHOs which are relevant for identification and measurement in a future questionnaire focusing on the EEs of PH interventions. In general, experts mentioned NHOs on all levels, but measurement on a direct social level was mentioned less often in comparison with measurement at the individual or societal level. However, it is not possible to conclude from this that the experts find the direct social level less important. In addition, as the experts did not mention the different NHOs on the various levels much in detail, but in more general terms, this can also be a reason that there are fewer quotes in the ‘’direct social” subtheme.

This study confirms the findings of two other studies within our research group - one investigating the view of the Dutch public in general [30], and another, a systematic review on the use of NHOs for the evaluation of PH interventions in the Netherlands [van Mastrigt GAPG, Alayli-Goebbels AFG, Lawson KD, Evers SMAA. Identifying non-health outcomes of health promotion for consideration in economic evaluations from a societal perspective *Submitted*]. International experts acknowledge similar NHO topics to be important. Eight out of the ten most frequently used NHOs of evaluated PH interventions described in the systematic review [>van Mastrigt GAPG, Alayli-Goebbels AFG, Lawson KD, Evers SMAA. Identifying non-health outcomes of health promotion for consideration in economic evaluations from a societal perspective Submitted] were also mentioned headings by our experts. These NHO headings were, respectively, “self-confidence”, “(un) healthy behavior”, “perceived life control”, “social life”, “educational achievements”, “employability”, “participation and integration”, and “justice and security” [30]. Two others mentioned by Benning et al., “knowledge about a certain health problem” and “relaxation” were not frequently mentioned by the experts. The first of these two subthemes was categorized in our study as part of the heading educational achievement and the second one or related terms were not mentioned at all by any of our interviewed experts.

The use of three different perspectives in the three previously discussed studies (our study and [30] [van Mastrigt GAPG, Alayli-Goebbels AFG, Lawson KD, Evers SMAA. Identifying non-health outcomes of health promotion for consideration in economic evaluations from a societal perspective Submitted]) resulted in three different lists of NHOs which contain similar NHO domains and topics. These three lists form the basis for further research in which a final list or theoretical framework can be developed. Further research should focus on developing clear definitions of the various NHOs and investigating the relations between them. Subsequently, this may also imply a potential overlap between the NHOs which needs to be identified; the categories under which these NHOs fall need to be mapped.

### Methods for measuring and -valuating NHOs

Some of the experts discuss the use of existing questionnaires for measuring broader outcomes. However, these instruments mainly focus on an individual level, but there are also firm reasons to focus on higher levels, specifically on the direct social and societal levels as well [31, 32]. Furthermore, it is also important to investigate how the general public feels about using particular measures of personal well-being in order to inform policy decisions. They mentioned three types of existing questionnaires as alternative methods for measuring broader outcomes: the Well-being, Happiness [31, 33] and Capability Indices. Examples of the latter are the ICEpop CAPability measures, ICECAP-A for Adults [12], ICECAP-O for Older people [13] and ICECAP-SCM for use in end of life settings [14]. The capability indices are preference-based measures and were specifically designed to measure what individuals are able to do or, in other words, are ‘’capable of” [34, 35]. Various single and multiple item questionnaires for measuring well-being and happiness exist, including the General Health Questionnaire, (GHQ), the Orientations to Happiness Scale and the Satisfaction With Life Scale (SWLS); their outcomes were compared in the report by Dolan et al. [31].

However, as the above mentioned instruments focus mainly on an individual level, there are also firm reasons to focus on the direct social and societal levels as well [31, 32]. A questionnaire that is able to measure more levels of NHOs at once does not yet exist. The views of the experts give enough clues to start developing a new instrument which can be used for measuring NHOs in economic evaluations. The challenge will be to develop a questionnaire which meets all, sometimes contrasting, requirements at once. It will not be possible to develop a questionnaire that is broad, sensitive to change, includes all NHO levels, and is also short and manageable. In our view, to keep the burden for the respondents as low as possible, the instrument should employ a modular system. This can contain different items per NHO domain, from which the researcher is able to pick whatever he finds relevant for him/her to use in evaluating that particular PH intervention. Therefore we should start with the identification and measurement of individual and direct social level NHOs, because we think this is most feasible. We also agree with some of the experts that in the design phase of a new questionnaire, the -valuation aspects of NHOs is a very important issue which needs to be taken into account. In addition, a discussion on how the NHOs can be incorporated into current economic evaluation methodology needs to be considered, as otherwise it will be difficult to inform decisions on the reimbursement of new public health interventions, based on the outcome of the questionnaire.

### Alternative methods to value NHOs

For EEs of complex PH interventions, it’s difficult to evaluate the use of the preferred framework RCT, CEA and QALY, as randomization is sometimes not ethical (e.g. PH interventions on the effects of tobacco smoke) or difficult due to practical reasons (e.g. structural PH interventions). However, not including NHOs but only health outcomes (QALYs) in the evaluation of PH intervention can lead to wrong or poor decisions because the full picture of potential effects or benefits is not taken into account. Therefore, alternative methods for -valuation of PH interventions, besides the traditional CEA and CUA framework, are needed; several options for this will be discussed in more detail in the next section. First, the CBA (which expresses both costs and outcomes in monetary values), although frequently mentioned by experts, it is not often used in the EE of PH interventions [2–4]. A reason for this can be that there is some doubt regarding the internal and external validity of using this type of stated preference methodology, due to numerous practical and methodological problems [36, 37]. These are, for instance, the lack of standardisation of elicitation methods (willingness to pay or discrete choice experiments), and some of the NHOs (like self-esteem or improvement in education) are very hard to express in monetary values. In addition, as a CBA needs to be tailored for every specific intervention, the measurement burden is substantially increased if the CBA is used together with other health-related outcome measures. Furthermore, stated preference questions are prone to biases, like starting point biases and range biases [38].

Second, the CCA was also mentioned as alternative method. All relevant outcomes can be taken into account In this type of EE, but because it generates no generic overall outcome measure, it is difficult to compare across different types of interventions [2]. Despite this drawback, the CCA seems to very helpful for the economic evaluation of complex PH interventions [39] and for informing the needs of different end users of economic evidence: the funder, the makers of an agenda for public health policy and the social welfare agenda [40]. Currently, the CCA is not a popular method for evaluating PH intervention; the reason for this can be that is difficult to determine the overall impact of the evaluated intervention when some outcomes improve and others get worse [41].

Third, using CUA together with a ‘’broader QALY” as an alternative method was also mentioned by experts. This can be done, for example, when HrQoL is measured using the EuroQoL, which contains five dimensions: mobility, self-care, usual activities, pain/discomfort, anxiety/depression and adding one other dimension, such as cognition, if this is relevant for evaluation purposes. The utility calculated using this method results in a broader QALY than if the extra dimension has not been taken into account.

Fourth, the MDCA, which takes multiple criteria, like cost-effectiveness, budget impact, severity of illness, affordability and accessibility of health care into account, was also cited by some experts as an alternative method [42]. The disadvantage of this method is that the methodology is still developing and a standardized approach is not yet available [43]. The CUA using the broader QALY, the CCA and the MCDA received relatively little mention by the experts, indicating either that not all experts are familiar with these methods or they don’t think these are the preferred methods for tackling this measurement problem.

This also accounts for the fifth possibility for the -valuation of NHOs; a CEA or CUA using a multi-sectorial approach or an inter-sectoral compensation test. With this method, the inter-sectoral costs and benefits are simultaneously captured and adjusted for budgets and resources that are allocated by various ministries [18]. EEs of PH interventions often require such a multi-sectoral collaboration [3, 44] as not only outcomes generated in the healthcare sector, but also gains and losses generated in other sectors, such as education, crime and justice and the employability sector need to be taken into account. A recent NICE guideline (PH50) states that future research should address this topic: ‘’There is a lack of high quality studies measuring the effects of multi-faceted and multi-sectoral approaches to the prevention of domestic violence” [9].

One of the topics not discussed by the experts is the use of social return on investment (SROI) methods which can be used by local authorities to determine the cost-effectiveness of specific intervention [45]. They may be important in the EEs of PH interventions, particularly in determining what NHOs are relevant for each of the stakeholders.

### Other methodological challenges of EEs of public health interventions

In the previous sections, various methodological challenges are mentioned in relation to NHOs and PH interventions. However, three other relevant methodological challenges were identified from the literature. Although these are not the subject of the current study, they provide the reader a full picture of other important issues. Therefore, they will be briefly discussed as well. First, in general the time horizon of PH studies needs to be longer than for most of the effectiveness studies, as the effects don’t always occur within 1 or 2 years. Ideally these effects would include lifetime costs and the consequences of the PH interventions being evaluated [46]. Second, in relation to the design of PH interventions, for the most part they are tailored to the local situation, meaning they are not standardized, making comparison between different types of settings very difficult. Finally, reducing health inequities is one of the important objectives for PH interventions; however, these are normally not incorporated in the EEs of these interventions [47].

### Limitations

The current study was subject to several limitations. First, we included mainly scientific experts working in academia, and selected no other stakeholders who may have different views on this topic. However, as mentioned in the result section, 10 of the experts were members of the advisory councils involved in PH decision-making and 6 of them worked in governmental institutions performing research which generates evidence for informing policy decisions. Aside from this limitation, we think we have interviewed experts with considerable experience and a thorough knowledge of the study topic. As noted in the result section, on average our experts have 22 years of work experience; their knowledge is also indicated by the fact that they mentioned more than 300 NHOs in total.

A second limitation of our study might be the limited operationalisation of the different NHO terms, as the experts discussed NHOs in general terms. In other words, this qualitative research resulted in a limited level of detail in the description of NHOs in comparison with quantitative studies especially designed to measure NHOs in an EE of a specific PH intervention.

Third, between the two researchers who performed the coding, some disagreement was observed in coding the interviews. This was related to the difference in the level of detail in the initial coding by one of the two researchers. However, in consensus meetings with the various authors any discrepancies were discussed and resolved.

Fourth, the expert interviews were held more than 3 years ago. While it is possible that some of the experts’ views have changed in light of new evidence, we do not expect considerable changes in their views, as no experts expressed a wish to change the description of their views when they read the transcripts of interviews as we started to write this manuscript. In addition, recent publications highlight the importance of considering NHOs in the evaluation of mental health interventions and interventions to manage overweight and obesity, which were also identified in this study (e.g. self-esteem) [10, 48].

Fifth, as already noted, our definition of the broader outcomes was a research restriction. We defined non-health outcomes as all outcomes that were not measured using the EQ-5D. While this definition is in line with common economic evaluation practice, current health outcomes could also be defined in a broader fashion by using broader outcome instruments such as the capability indices as a reference [34].

Finally, some of the NHOs mentioned by experts, such as changes in lifestyle behaviors (e.g., an increase in sport activities or reduction of alcohol use), defined in our study as (un) healthy behaviors, are included as effectiveness measures of the PH interventions in the economic evaluation studies.

## Conclusions

Leading experts indicate that there is a very broad field of NHOs on all three levels – individual, direct social, and societal - which can be relevant in measuring the outcomes of public health interventions, and a majority of experts are in favor of developing a new instrument to identify and measure broader outcomes in the economic evaluations of public health interventions. Future research needs to focus on instrument development, addressing the challenges raised by multiple conflicting requirements. In addition, an open discussion needs to be started with all stakeholders regarding which steps need to be taken for future research on this topic.

## Appendix 1

### Interview protocol INFORMEH

First of all, would you mind if I tape the interview with this voice recorder, because that would make it easier for me to summarize the results afterwards?

Let me start with a short introduction of the INFORMEH project and the aim of the interview. The INFORMEH project is a two-year project and I started working on it a couple of months ago at Maastricht University. The aim of the project is to identify relevant broader non-health outcomes of health promotion, which are currently neglected in economic evaluation studies. To give you an idea about which non-health outcomes we are thinking about I will give you some examples. You can think, for example, of outcomes that are relevant for the individual him or herself, such as increased self-esteem among elderly people who participate in a falling prevention program. But one can also think of effects that are relevant for others, such as the reduction of domestic violence among family members of addicted people who are in a rehab program. Ultimately we want to develop an instrument that makes it possible to measure such non-health outcomes and incorporate these in economic evaluation studies. Because there is little scientific literature on which outcomes could be relevant, we decided to organize interviews with experts such as you. So I hope this gives a short impression about what the INFORMEH project is about; do you have any questions so far?

Show EQ5D if necessary

Q1: To start with, could you please tell something about your work and the field you’re working in? (Let the expert make himself known, gives possibility to gather impressions about possible important non-health outcomes (in his field))

Q2: Do you have experience with economic evaluations or health promotion? And if so, could you tell me something about what kind of interventions you have evaluated? (Let him talk about outcomes that are important from his perspective, how much he knows about the topic of, making him thinking of gaps and missing but important things)

Q3: Do you know of any interventions in the field of health promotion that intentionally or unintentionally yielded broader, non-health outcomes? (deeper insight)

If yes, ask additional questions on if / how these outcomes were measured.

If no, ask additional questions on interventions outside the field of health promotion.

Q4: Do you know of any effect studies in the field of health promotion that included such outcomes?

If yes, ask additional questions on how these outcomes were measured.

If no, ask additional questions on effect studies outside the field of health promotion.

Q5: Do you know of any economic evaluation studies in the field of health promotion that included such outcomes?

If yes, ask additional questions on how these outcomes were measured.

If no, ask additional questions on economic evaluation studies outside the field of health promotion.

Q6: According to you, what are the most important outcomes domains of health promotion initiatives, either in terms of health, or broader, non-health outcomes, and either intended or intended outcomes? (after having him made alert on important outcomes, now letting him rank or summaries the importance of them)

Q7: How should an instrument for measuring non-health outcomes for the economic evaluation of health promotion look, in your opinion? (does he know what the meaning is of an instrument, knows other instruments?)

- Which domains should be included?
- At what level should we measure non-health outcomes: individual, direct social environment or society as a whole?
- Which requirements should such an instrument have to meet?

Q9: In your opinion, what are the main future challenges for measuring non-health outcomes in economic evaluations of health promotion?

Q10: Are there any topics that haven’t been addressed during this interview, but that you consider useful with regard to this research theme?

These were all my questions, thank you for your input, do you have any questions?

Regarding the remainder of my project, I will use these interviews as input for a paper which will present a theoretical framework about non-health outcomes in economic evaluations. Besides, we are also planning a Delphi panel in the near future in which we further try to select the most relevant outcomes for economic evaluations of health promotion. It would be great if you were willing to serve as an expert member of that panel.

## Appendix 2

### Coding themes

Non-health outcomes (NHOs): outcomes not captured by the five dimensions included in the EQ-5D (mobility, self-care, usual activities, pain/discomfort and anxiety/depression).

(1.1) Individual level: all NHOs that focus on individuals targeted in the PH interventions.
(1.2) Direct social environment: all NHOs that indirectly influence individuals; these people do not participate themselves in PH interventions but whose relatives, classmates or spouses do participate.
(1.3) Society as a whole: all NHOs that have an impact on society. For instance: crime, employment in general, education in general.
(2.1) Views: views related to the usefulness and of the INFORMEH instrument and quotes on NHOs in general.
(2.2) Requirements: views related to the content and valuation of the INFORMEH instrument, such as levels, domains, valuation issues and also feasibility.
(3.1) CBA: quotes on the use of Cost Benefit Analysis for the measurement and valuation of NHO, such as WTP.
(3.2) Happiness index: quotes on the use of the Happiness index or Well-being index for the measurement and valuation of NHOs
(3.3) Capability approach quotes on the use of the Capability Approach for the measurement and valuation of NHOs, (including ICECAP).
(3.4) Broaden the QALY concept: quotes on the use of the Broaden the QALY concept for the measurement and valuation of NHOs, such as Super-QALY and societal QALY. ASCOT
(3.5) Other alternatives: quotes on the use of alternative methods (those not defined as CBA, Happiness Index, Capability approach, Broaden QALY concept), e.g. MDCA, CCA, and the multi-sectoral approach for the measurement and valuation of NHOs.
(4.0) General views about health promotion/economic evaluations: all other general quotes on PH and EE.

### Changes to initial coding

First version of definitions made by MJA before the start of coding by LS

The definitions are discussed by group (GvM, AP, SE and AG at 24–6 2013).

The following adaptions were done;

1. The word “Impact” in definition 1.1.,1.2,1.3 deleted
2. ‘’Targeted interventions” is added in cat. 1.1 and 1.2
3. 2.1 included views on usefulness and general quotes on NHOs (before coded in 4.0)
4. 2.2 included quotes on feasibility (by LS before coded as 2.1) will be grouped in 2.2, as these mainly concern statements on the contents of the INFORMEH-instrument

The definitions are discussed by AP and GvM on 27–6 2013

1. Coding on feasibility by will be placed in 2.2 (GvM will recode these quotes, using find and search in NVivo, if mentioned in discussion on agreement in coding)
2. Education, crime and productivity in general are placed in 1.3; however, if the interventions are focused on individuals, these quotes are placed in 1.1.
3. Quotes on CCA are categorized in 3.5

Coding

Unclear (LS) and no coding (GvM): no coding of text part for final coding

Valuation of future challenge (LS): theme 4.0

Usefulness of the instrument (LS): theme 2.1

## Appendix 3

**Table 1:** Summary of the NHOs mentioned by the experts

NHOs	Heading	Example quotes
Individual level		
	Educational achievements	*‘’educational output”*
		*‘’reduction of school dropout”*
		*‘’literacy and numeracy skills”*
		*‘’an increase of Intelligent Quotient (IQ)”*
	Social life	*‘’frequency of social contact”*
		*‘’going out with friends*
	(Un) healthy behavior	*“’frequency of walking”*
		*‘’alcohol and or drug abuse”*
	Perceived life control	*‘freedom”*
		*‘’autonomy”*
	Emotions	*‘’a sense of satisfaction”*
		*‘’enjoyment”*
	Self-confidence	*‘’self-esteem”*
		*‘’self-efficacy”*
	Employability	*‘’ability to work”*
		*‘’being productive”*
	Family life	*‘’have a partner”*
		*‘’the ability to conceive”*
	Physical environment	*‘’insulation of houses”*
		*‘’pollution (asbestosis)”*
	Justice and security	‘*’new security mechanisms for houses”*
		*‘’contact with the police or the judicial system”*
	End of life aspects	*‘’being prepared for death‘’*
		*‘’emotional and physical suffering”*
	Use of medical treatment	*‘’medicine use”*
		*‘change of medical regime*
	Perceptions	*‘’their perception of crime and fear”’*
		*”changes in people's perception”*
	Other	*‘’activities at home”*
		*‘’sexual dysfunction”*
Direct Social level		
	(Un) healthy behavior	*‘’cooking and eating properly of the family”*
		*‘’reducing aggression and violence”*
	Educational achievements	*‘’increase of concentration levels of the class”*
		*‘’impact on truancy rates in classes”*
	Social life	*‘’Impact of social cohesion on neighborhood”*
		*‘’social exclusion”*
	Employability	*‘’absenteeism and presenteeism of caregivers”*
		*‘’the sickness-leave of teachers”*
	Well-being	*‘’Improved well-being in neighborhood’*
		*‘’effect of increase of well-being across generations’*
	Physical environment	*‘’how dwelling conditions (space, privacy) affect relations within family/ household”*
		*‘’accelerated damage due to damp houses to their children”*
	Perceptions	*‘’people's perception of their neighborhoods”*
		*‘’children’s perception of growing up in better environments”*
	Other	*‘’individual resilience impacts of family members”*
		*‘’innovation and creativity”*
		*‘’emotional burden on family and friends”*
Societal level		
	Labor participation and productivity	*‘’reduction of number of teachers”*
		*‘’lack of employment”*
	Justice and security	*‘’crime related to alcohol consumption”*
		*’impact of social security on society”*
	Unhealthy behavior	*‘’bullying”*
		*‘’drug (mis)use”*
	Use and availability of healthcare services	*‘’having antibiotics available”*
		*‘’use of social services and amenities”*
	Participation and connectedness	*‘’social connectedness”*
		*‘’sense of community”*
	Educational achievements	*‘’education outcomes”*
		*‘’educational attainment would improve educational achievements of children”*
	Transport	*‘’traffic injuries”*
		*‘’impact on congestion/traffic jams on the roads”*
	Economic	*‘’poverty”*
		*‘’financial exclusion”*
		*‘’impact on the local economy”*
		*‘’(social) welfare and effects”*
		*‘’social capital”*
	Physical environment	*‘’housing rates of homelessness”*
		*‘’damp housing”*
	Other	*‘’learning disability workers”*
		*‘’is interested in resilience in terms of coping with difficult situations”*
		*‘’Inequalities”*

## Abbreviations

NHOs: Non-health outcomes
EE: Economic evaluations
PH: Public Health
CEA: Cost-effectiveness analyses
CUA: Cost-utility analyses
CCA: Cost-consequence analysis
QALYs: Quality-adjusted life years
CBA: Cost benefit analysis
MCDA: Multi-criteria decision analyses
EQ-5D: EuroQoL
IQ: Intelligent Quotient
GHQ: General Health Questionnaire
SWLS: Satisfaction With Life Scale
